# Melanozytome: Intermediäre melanozytäre Neoplasien mit distinkter Morphologie und molekularem Hintergrund

**DOI:** 10.1007/s00292-026-01601-7

**Published:** 2026-10-15

**Authors:** Daniela Mihic-Probst, Francesca Saro

**Affiliations:** Institut für Pathologie und Molekularpathologie, Universität Zürich, Universitätsspital Zürich, Schmelzbergstr. 12, 8091 Zürich, Schweiz

**Keywords:** Unbekanntes Potential, Melanom, Nävus, Hochdurchsatz-Nukleotidsequenzierung, Protoonkogene Proteine BRAF, Unknown potential, Melanoma, Nevus, High-throughput nucleotide sequencing, Proto-oncogene proteins BRAF

## Abstract

Bereits in den 1990er-Jahren zeigte Vogelstein, dass die Onkogenese häufig durch eine initiale „Treibermutation“ ausgelöst wird, gefolgt von der Akkumulation weiterer genetischer Alterationen im Verlauf der Tumorprogression. Sowohl beim Nävus als auch beim Melanom ist *BRAF-V600E* oft diese Treibermutation, während zusätzliche „second hits“ das Progressionsrisiko zum Melanom bestimmen.

Die 5. Auflage der WHO-Klassifikation der Hauttumoren führt den Begriff der Melanozytome (MZ) ein. Zu den in diesem Beitrag behandelten MZ zählen WNT-aktivierte, tief penetrierende/plexiforme Melanozytome (DPN), pigmentierte epitheloide Melanozytome (PEM), BAP1-inaktivierte Melanozytome (BIM) sowie MITF-Signalweg-aktivierte melanozytäre Tumoren. Allen MZ gemeinsam ist eine intermediäre Biologie zwischen benignen Nävi und Melanomen mit geringem Progressionsrisiko, eine charakteristische Histopathologie sowie ein definierter molekularer Hintergrund. Dieser umfasst häufig eine *BRAF-V600E*- oder *MAP2K1*-Mutation sowie einen „second hit“ mit Aktivierung eines weiteren Signalwegs.

MZ werden der Gruppe 2 der Risikostratifizierung melanozytärer Neoplasien zugeordnet und sollten vollständig exzidiert werden. Die Abgrenzung zum Melanom kann teilweise schwierig sein. In solchen Fällen kann ein Sicherheitsabstand von bis zu 1 cm empfohlen werden. Bei Borderlineläsionen hinsichtlich der Dignität sind molekulare Zusatzuntersuchungen, z. B. mittels Next-Generation Sequencing, oft hilfreich. Hinweisend auf ein Melanom sind die homozygote *p16*-Deletion, die *TERT*-Promotormutation oder mehr als 6 chromosomale Kopienzahlveränderungen.

## Abstract (en)

As early as the 1990s, Vogelstein demonstrated that oncogenesis is often initiated by an initial “driver mutation,” followed by the accumulation of additional genetic alterations during tumor progression. In both nevi and melanoma, *BRAF V600E* frequently represents this driver mutation, while additional “second hits” determine the risk of progression to melanoma.

The 5th edition of the *WHO Classification of Skin Tumours* introduced the term melanocytoma (MZ). This group includes WNT-activated deep penetrating/plexiform melanocytomas (DPN), pigmented epithelioid melanocytomas (PEM), BAP1-inactivated melanocytomas (BIM), and MITF pathway-activated melanocytic tumors. All melanocytomas share an intermediate biological behavior between benign nevi and melanoma, with a low risk of progression, characteristic histopathological features, and a defined molecular background. This often includes a *BRAF V600E* or *MAP2K1 *mutation, along with a second hit involving activation of an additional signaling pathway.

Melanocytomas are classified within risk group 2 of melanocytic neoplasia stratification and should be completely excised. Distinguishing them from melanoma can be challenging in some cases; in such situations, a surgical margin of up to 1 cm may be recommended. In borderline lesions with uncertain biological potential, additional molecular analyses—such as next-generation sequencing—are often helpful. Findings suggestive of melanoma include homozygous *p16* deletion, *TERT* promoter mutation, or more than six chromosomal copy number alterations.

## Gesamtschau

Schon in den 1990er-Jahren zeigte Vogelstein, dass sich Krebs durch die schrittweise Ansammlung genetischer Veränderungen entwickelt [5]. Davies et al. wiesen 2002 die *BRAF*-V600E-Mutation im RAS-RAF-MEK-ERK-Signalweg als entscheidenden Treiber bei 66 % der Melanome nach [8]. Pollock et al. entdeckten jedoch, dass dieselbe Mutation auch in benignen Nävi häufig ist, sodass sie allein das Fortschreiten zum malignen Melanom nicht erklärt [19].

Dank neuer Technologien wie Next-Generation Sequencing (NGS) hat sich seither das molekulare Verständnis des Melanoms rasant weiterentwickelt. Unter Berücksichtigung der Klinik, der Histopathologie und molekularen Erkenntnisse sind in der 5. Auflage der WHO-Klassifikation Hauttumore 9 Entstehungswege des Melanoms dokumentiert [15].

Neben *BRAF-*V600E treten bei melanozytären Nävi weitere subtypenspezifische Treibermutationen auf: *NRAS* bei kongenitalen Nävi, *GNAQ/GNA11* bei blauen Nävi und Genfusionen oder *HRAS* bei Spitz-Nävi [21]. Molekulare Analysen melanozytärer Nävi mit distinkter Histopathologie zeigen, dass neben einer Treibermutation – häufig *BRAF-*V600E – oftmals eine zusätzliche sekundäre genetische Veränderung („second hit“) vorliegt. So weist beispielsweise der tief penetrierende Nävus („deep penetrating nevus“, DPN) neben der Treibermutation *BRAF-*V600E häufig eine Aktivierung des WNT-Signalwegs auf [29]. Diese und weitere Läsionen wurden in der WHO-Klassifikation neu als Gruppe der Melanozytome (MZ) aufgenommen.

MZ gelten als intermediäre Läsionen im biologischen Kontinuum zwischen benignem Nävus und Melanom. Sie werden dem Risikoprofil für melanozytäre Tumoren der Gruppe 2 zugeordnet, da das Progressionsrisiko insgesamt gering ist [1]. MZ sollten grundsätzlich vollständig im Gesunden exzidiert werden. Ein Sicherheitsabstand sollte den Fällen vorbehalten bleiben, in denen ein Melanom histologisch und dann auch in der klinisch-pathologischen Korrelation nicht sicher ausgeschlossen werden kann. Je nach Progressionsrisikoeinschätzung wird ein Sicherheitsabstand von 2 mm bis 1 cm betragen [11].

Spitz-Melanozytome werden in dieser Übersicht nicht behandelt, da sie durch genetische Progression aus einem Spitz-Nävus hervorgehen und dem eigenständigen Spitz-Spektrum zugeordnet werden. Aufgrund morphologischer und teilweise molekularer Überschneidungen sind Spitz-Tumoren jedoch differentialdiagnostisch relevant und werden in den entsprechenden Abschnitten berücksichtigt [10, 26].

## WNT-aktiviertes, tief penetrierendes/plexiformes Melanozytom (DPN)

Die Erstbeschreibung des DPN erfolgte 1989 durch Seab et al. Das molekulare Profil des DPN mit kombinierter Aktivierung des MAP-Kinase- und des β‑Catenin-Signalwegs wurde jedoch erstmals 2017 von Yeh et al. publiziert [22, 29].

### Pathogenese

DPN entstehen häufig auf dem Boden eines konventionellen Nävus und tragen meist *BRAF-*V600E*- *oder *MAP2K1*-Mutationen. Makroskopisch und histopathologisch ist manchmal eine konventionelle Nävuskomponente erkennbar. Seltener finden sich *HRAS-*Mutationen oder *BRAF-*Fusionen. Charakteristisch ist stets die Aktivierung des WNT-Signalwegs, überwiegend durch *CTNNB1*-Mutationen in Exon 3, selten durch *APC-*Verlust [29].

### Klinik

Das mittlere Erkrankungsalter liegt bei ca. 30 Jahren, Frauen sind leicht häufiger betroffen. Läsionen können jedoch in jedem Alter auftreten. Bevorzugte Lokalisationen sind Kopf-Hals, gefolgt von Extremitäten und Stamm; auch die bulbäre Konjunktiva, Plica und Karunkel können betroffen sein [24].

### Makroskopie

Der DPN ist eine symmetrische, scharf begrenzte, dunkel pigmentierte Papel. Bei dominanter konventioneller Nävuskomponente kann sich eine dunkle Papel innerhalb einer pigmentierten Makula zeigen, wodurch aufgrund der Mehrfarbigkeit und relativen Asymmetrie klinisch Malignitätsverdacht aufkommen kann.

### Histologie

**Abb. 1:**
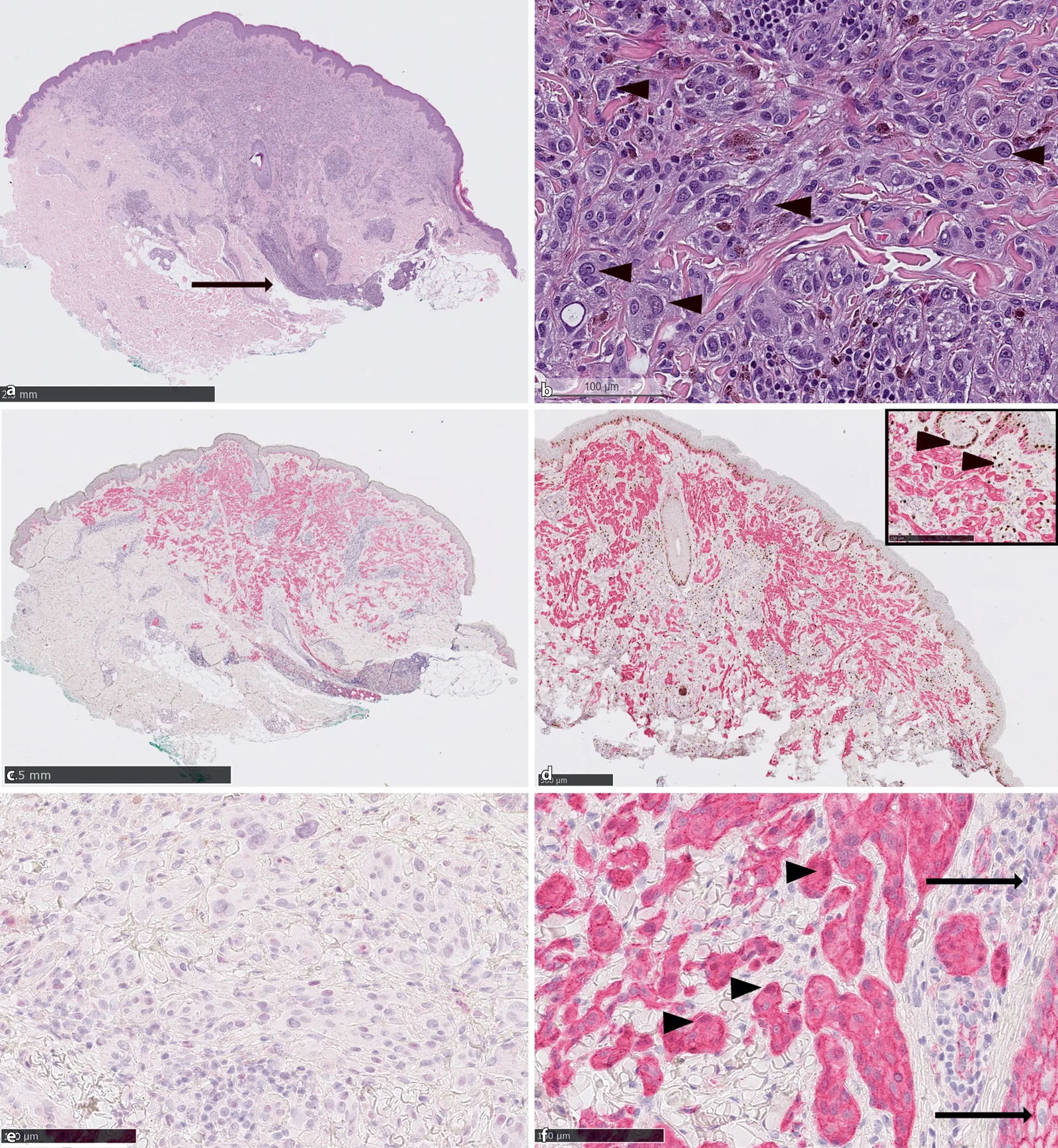
WNT-aktiviertes, tief penetrierendes Melanozytom. **a** HE Übersicht: Dermale bis tief subkutan reichende Läsion mit Vorwölbung der Epidermis. Dermales keilförmiges teils faszikuläres Wachstum (*Pfeil*; Vergr. 25:1). **b** Vergrößerung von **a**: Ausgeprägte Zellkernpolymorphie, teils deutlich atypische epitheloide Zellen mit prominenten Nukleolen (*Pfeilspitzen*), eingestreute Melanophagen und Kollagenbänder (Vergr. 400:1). **c** Positivität für HMB45 in gesamter Läsion (Vergr. 25:1). **d** Melan‑A (*rot*)/Ki67 (*braun*): Proliferation < 1 % (Vergr. 100:1). *Inset*: proliferierende Keratinozyten und Leukozyten (*Pfeilspitze*). **e** BRAF sehr schwache, fragliche Positivität (Vergr. 200:1). **f** β-Catenin: Nukleäre Positivität in Melanozyten (*Pfeilspitzen*) bei Negativität in Keratinozyten und Leukozyten (*Pfeile*; Vergr. 200:1)

**Abb. 2:**
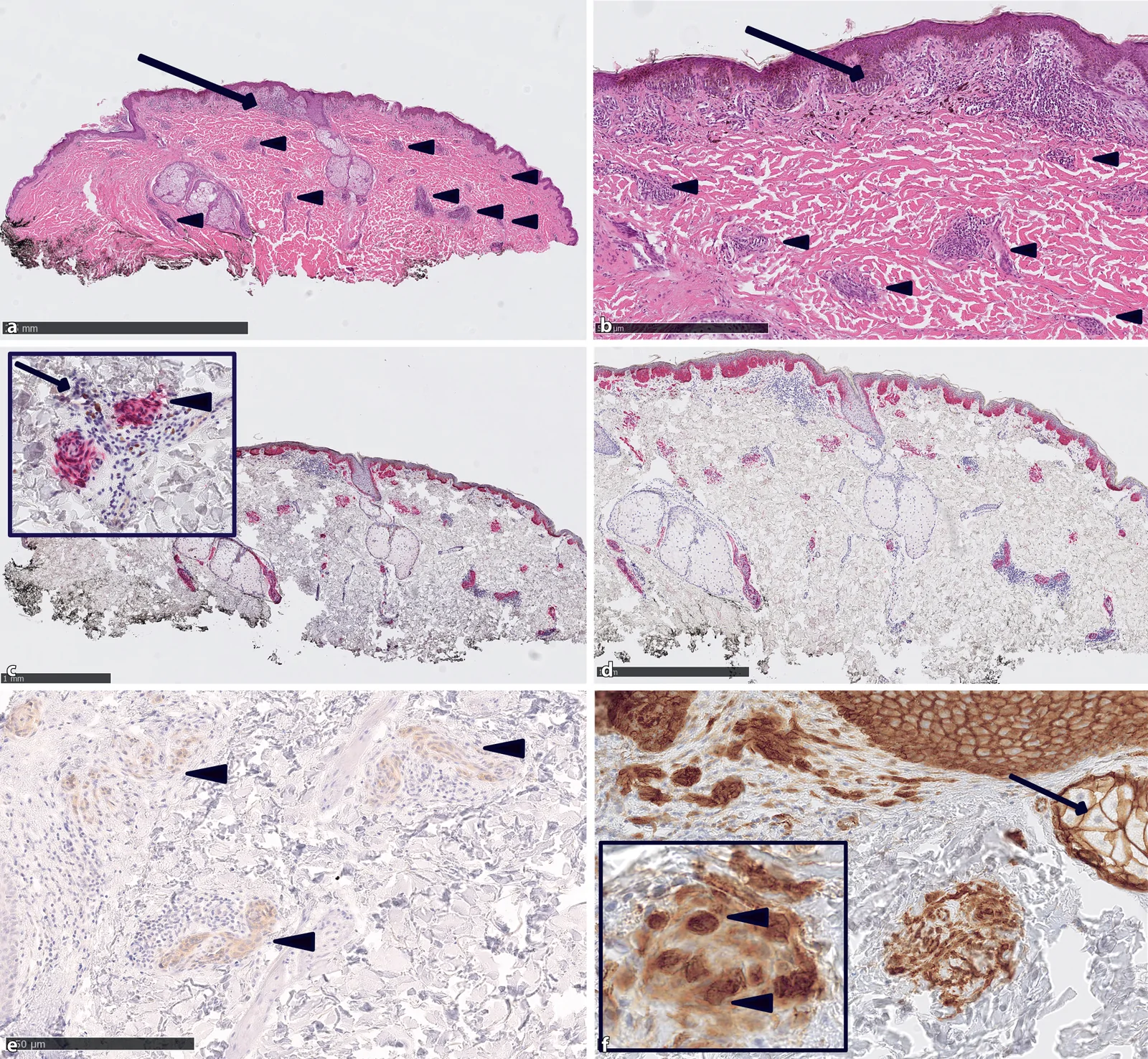
WNT-aktiviertes plexiformes Melanozytom.** a** HE-Übersicht: Oberflächlich dermales Wachstum entlang von neurovaskulären Strukturen und Adnexen (*Pfeilspitzen*; Vergr. 25:1). Geringe junktionale Komponente (*Pfeil*). **b** HE-Vergrößerung von **a**: Junktionale Zellnester (*Pfeil*) und plexiformes Wachstum entlang neurovaskulärer Strukturen (*Pfeilspitzen*; Vergr. 150:1). **c** Doppelfärbung Melan‑A (*rot*)/Ki67 (*braun*): Proliferation < 1 % (Vergr. 25:1). *Inset*: Melan‑A positive Zellen ohne und Entzündungszellen mit Proliferation (*Pfeilspitze*). **d** HMB45: Diffus positiv (Vergr. 50:1). **e** BRAF-Negativität (mit NGS bestätigt) und Nachweis einer *MAP2K1*-Mutation (Vergr. 200:1). **f** β-Catenin nukleär positiv in melanozytären Zellen (*Pfeilspitzen*), negativ in Talgdrüse (*Pfeil*; NGS-Nachweis einer *CTNNB1*-Mutation; Vergr. 300:1)

Die unterschiedliche Terminologie spiegelt 2 verschiedene Wachstumsmuster wider:

- Tief dermal reichend, keilförmig, bulböse/faszikuläre Invasionsfront, oft mit junktionaler Komponente; Zellen spindel- bis epitheloidzellig, doppelt so groß wie basale Keratinozyten. Feingranuläres Pigment, niedrige Mitoserate (≤ 2 Mitosen/mm^2^). Zwischen den locker gelagerten Zellen finden sich eingestreute Kollagenfasern sowie zahlreiche Melanophagen, sodass ein „schachbrettartiges“ Muster vorliegt (Abb. 1).
- Plexiform, oberflächlich dermal, faszikulär-plexiform entlang neurovaskulärer Strukturen, häufig mit konventioneller Nävuskomponente (Abb. 2).

### Immunhistochemie

Die gesamte Läsion zeigt eine diffuse Positivität für HMB45 ohne die für konventionelle Nävi typische reifungsabhängige Abnahme der Expression in der Dermis. Aufgrund der Aktivierung des WNT-Signalwegs besteht eine nukleäre Positivität für β‑Catenin; auch LEF1 ist als Surrogatmarker in der Regel positiv. p16 zeigt einen Wildtyp-Expressionsstatus, Prame ist negativ (Abb. 1 und 2).

### Differenzialdiagnosen

Klinisch relevant ist vor allem das primäre oder metastatische Melanom. Histopathologische Kriterien sind meist wenig hilfreich. Hinweise auf Malignität sind ausgeprägte Entzündung, starke Atypien oder vermehrt Mitosen (> 2 Mitosen/mm^2^). Immunhistochemische und molekulare Analysen zeigen jedoch signifikante Unterschiede. Hinweisend auf Malignität sind: vollständiger p16-Verlust, positive Prame-Expression, *APC*-Mutation sowie ≥ 1 pathogene Zusatzmutation, insbesondere im TERT-Promotor [14]. Framedeletionen von *MAP2K* sind jedoch hinweisend für DPN [4].

Nukleäre β‑Catenin- und LEF1-Positivität hilft, DPN von zellreichen blauen und kombinierten Nävi abzugrenzen. Echte Spitz-Nävi müssen definitionsgemäß *BRAF*-Wildtyp sein [20].

### Prognose

DPN hat eine exzellente Prognose. Eine maligne Progression ist selten. Bei 355 klassischen und 48 Borderline-DPN in der Literatur wurden nur 5 Rezidive und 3 Metastasen berichtet, alle bei Borderline-DPN [7]. Der DPN wird daher als Gruppe II der melanozytären Tumoren klassifiziert [1]. Die gemeinsame Expertengruppe von ESP, EORTC und EURACAN empfiehlt für niedriggradige intermediäre melanozytäre Tumoren einen klinischen Resektionsabstand von 2 mm. Bei bereits vollständig exzidierten Läsionen mit freien, wenn auch knappen Rändern ist keine Nachresektion bis zu einem arbiträren Mindestabstand erforderlich, für hochgradige Läsionen werden dagegen 5–10 mm empfohlen. Kann ein Melanom nicht sicher ausgeschlossen werden, sollte die Behandlung entsprechend den Melanomleitlinien erfolgen [11].

## Pigmentiertes epitheloides Melanozytom (PEM)

Der Begriff PEM wurde 2004 von Zembowicz et al. eingeführt. Die Autoren zeigten, dass PEM, das aus stark pigmentierten epitheloiden Zellen besteht, dieselbe Morphologie aufweist wie epitheloide blaue Nävi von Patienten mit Carney-Komplex sowie dem Animal-type-Melanom. Sie wiesen dieselbe sporadische PRKAR1A-Inaktivierung nach, welche als Keimbahnmutation beim Carney-Komplex vorliegt [2, 31]. Aus diesem Grund führten sie den Begriff pigmentiertes epitheloidzelliges Melanozytom (PEM) ein [30].

Diese Erstbeschreibung legte den Grundstein für das heutige Verständnis des Begriffs Melanozytom als intermediärgradigen melanozytären Tumor mit distinkter molekularer Alteration und begrenztem metastatischem Potenzial. Aufgrund des in der Regel günstigen klinischen Verlaufs sollte der Begriff *Animal-type-Melanom* nicht mehr verwendet werden.

### Pathogenese

- Neben einer oder mehreren aktivierenden Mutationen im MAPK-Signalweg weisen etwa zwei Drittel der Läsionen eine inaktivierende *PRKAR1A*-Mutation auf; bei etwa einem Drittel findet sich eine* PRKAR1A*-Fusion [6, 16].
- PEM treten sporadisch oder im Rahmen eines Carney-Komplexes auf. Patienten mit Carney-Syndrom haben neben epitheloidzelligen blauen Nävi auch Myxome sowie verschiedene endokrine Neoplasien.
- Aktuelle Arbeiten zeigen, dass PEM trotz gemeinsamer PRKAR1A-Inaktivierung keine einheitliche Entität darstellen, sondern eine komplexe Gruppe sind. Abhängig von der Aktivierung des MAPK-Signalwegs werden 3 molekulare Subtypen berichtet: 1. Common-Nävus-assoziierte Gruppe mit *BRAF*-V600-Mutation. 2. Spitz-assoziierte Gruppe mit *HRAS*- oder *MAP2K1*-Mutationen bzw. -Fusionen. 3. Blue-Nävus-assoziierte Gruppe mit *CYSTLTR2*- oder *GNAQ*-Mutationen [10].

### Klinik

PEMs sind langsam wachsende, stark pigmentierte Papeln oder Knoten bei jungen Erwachsenen (medianes Alter 20–27 Jahre), können jedoch in jedem Alter auftreten (0,6–78 Jahre). Die Läsionen zeigen eine breite anatomische Verteilung, wobei Kopf, Hals und Extremitäten am häufigsten betroffen sind. In seltenen Fällen ist auch eine mukosale Manifestation möglich [30].

### Histologie

PEM sind überwiegend dermal lokalisierte Läsionen. Die Epidermis häufig leicht vorgewölbt und hyperplastisch. Die Zellen epitheloid und/oder dendritisch und stark pigmentiert. Diese bilden kompakte Zellverbände und Nester. Dem DPN ähnlich können sich die Tumorzellen auch um Adnexstrukturen der Haut sowie neurovaskuläre Bündel anordnen.

**Abb. 3:**
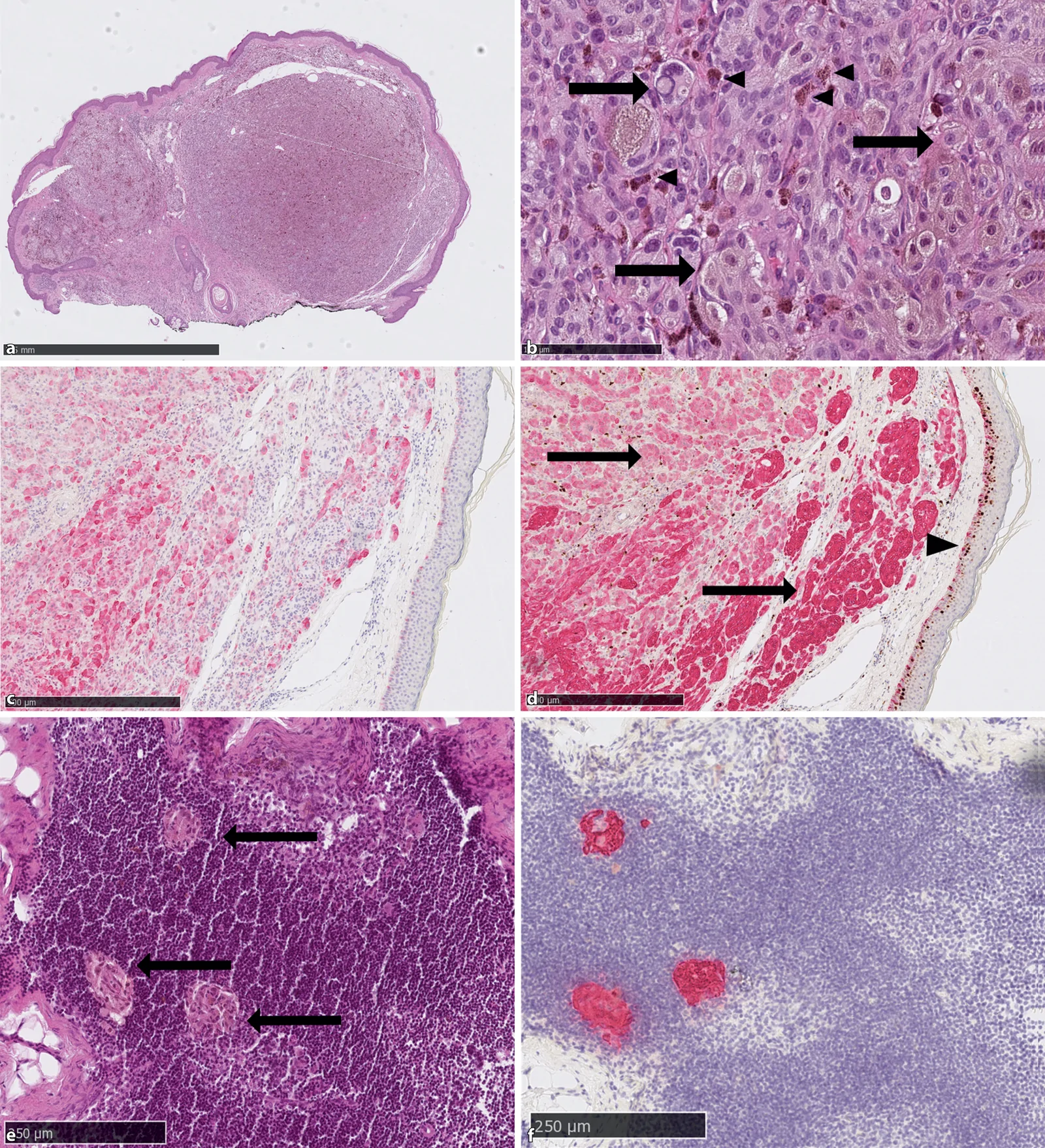
Pigmentiertes epitheloides Melanozytom (PEM). **a** Übersicht HE: Dermale melanozytäre Läsion, nodulär, expansiv, bulbös wachsend, Vorwölbung der Epidermis ohne Infiltration derselben oder adnexaler Strukturen (Vergr. 25:1). **b** Vergrößerung von **a**: Deutlich pigmentierte, große, epitheloide Zellen mit vesikulären runden Kernen und deutlichen Atypien (*Pfeile*). Eingestreut zahlreiche Melanophagen (*Pfeilspitzen*; Vergr. 200:1). **c** HMB45 diffus positiv (Vergr. 50:1). **d** Doppelfärbung Melan‑A (*rot*)/Ki67 (*braun*): Proliferation in Tumorzellen < 1 % (*Pfeile*); Positivkontrolle in Keratinozyten (*Pfeilspitzen;* Vergr. 50:1). **e** und **f** Nachweis einer Lymphknotenmetastase von (**a**): HE-Färbung, Vergr. 100:1 (**e**), Pan-Melanoma-Immunhistochemie, Vergr. 100:1 (**f**)

Zwischen den Tumorzellen finden sich zahlreiche stark pigmentierte Melanophagen, die in ein mehr oder weniger sklerotisches Stroma eingebettet sind. Die Invasionsfront bulbös oder invertiert keilförmig. In etwa 30 % der Fälle Nachweis einer diskreten junktionalen Komponente. Die Zellkerne sind meist vesikulär mit prominenten Nukleolen. Häufig finden sich mehrkernige Zellen, teilweise mit Reed-Sternberg-ähnlicher Morphologie (Abb. 3).

### Immunhistochemie

Ein immunhistochemischer Verlust von PRKAR1A liegt in 80 % der Fälle vor. PEMs können deutliche Atypien und Pleomorphie sowie Mitosen aufweisen. Sind diese ausgeprägt, kann der Begriff atypisches PEM verwendet werden. Manchmal ist die Abgrenzung zum Melanom unmöglich. Hinweisend auf ein Melanom ist ein immunhistochemischer Verlust von p16, eine TERT-Promotermutationen, chromosomale Kopienzahlveränderungen oder eine hohe Tumormutationslast [6].

### Prognose

Im Gegensatz zu den übrigen MZ weist das PEM ein höheres intermediäres malignes Potenzial auf. Regionale Lymphknotenmetastasen werden in bis zu 46 % der Fälle beobachtet, insbesondere bei ulzerierten Läsionen. Eine Fernmetastasierung über die regionalen Lymphknoten hinaus ist jedoch äußerst selten.

Selbst bei Vorliegen von Lymphknotenmetastasen ist die Prognose in der Regel exzellent [18, 30]. Das Management besteht aus kompletter chirurgischer Exzision mit engmaschiger Nachsorge.

## BAP1-inaktiviertes Melanozytom (BIM)

Die *BAP1*-inaktivierende Keimbahnmutation wurde erstmals 2011 von Wiesner beschrieben. Er dokumentierte 2 Familien mit multiplen atypischen epitheloiden Nävi. Einige Familienmitglieder entwickelten zudem kutane und uveale Melanome [27].

Nachfolgende Publikationen zeigten, dass die meisten BIM sporadisch sind und nur ca. 12 % eine Keimbahnmutation haben [26].

### Pathogenese

BIM treten in ~ 90 % auf dem Boden eines konventionellen Nävus mit *BRAF-V600E-*Mutation auf. Seltener finden sich *NRAS*-Mutationen oder *RAF1*-Fusionen. Der „second hit“ liegt in der Inaktivierung des Tumorsuppressorgens BAP1. Diese ist in ~ 73 % monoallelisch und in18 % biallelisch durch Loss-of-Function-Mutationen, Deletionen oder eine Kombination beider Mechanismen.

### Klinik

Patienten mit BAP1-Keimbahnmutation haben ein erhöhtes Risiko für verschiedene Malignome, wobei die Liste assoziierter Tumoren kontinuierlich erweitert wird. Besonders erhöht ist das Risiko für:

- *Uveale Melanome *(24–36 %). Die Läsionen treten meist sporadisch auf, können aber im Rahmen des autosomal-dominant BAP1-Tumorprädispositionssyndroms vorkommen. Patienten mit sporadischen BIM haben ein medianes Alter von 39 Jahren. Beim BAP1-Tumorprädispositionssyndrom sind die Patienten jünger (medianes Alter 25 Jahre) und haben multiple BIM (median 5 Läsionen).
- *Kutane Melanome* (17–36 %),
- Mesotheliome (14–24 %)
- Nierenzellkarzinome (11 %).
- Darüber hinaus werden erhöhte Inzidenzen von Pankreaskarzinomen, Meningeomen, Mammakarzinomen und Cholangiokarzinomen berichtet [25].

### Makroskopie

BIMs sind 4–12 mm große, hautfarbene bis rötlich-braune Papeln oder Knötchen. Sie können innerhalb eines konventionellen Nävus entstehen und aufgrund von Mehrfarbigkeit und Asymmetrie klinisch suspekt erscheinen. Die Läsionen treten bevorzugt an Kopf-Hals-Region, Rumpf und oberen Extremitäten auf [12].

### Histologie

**Abb. 4:**
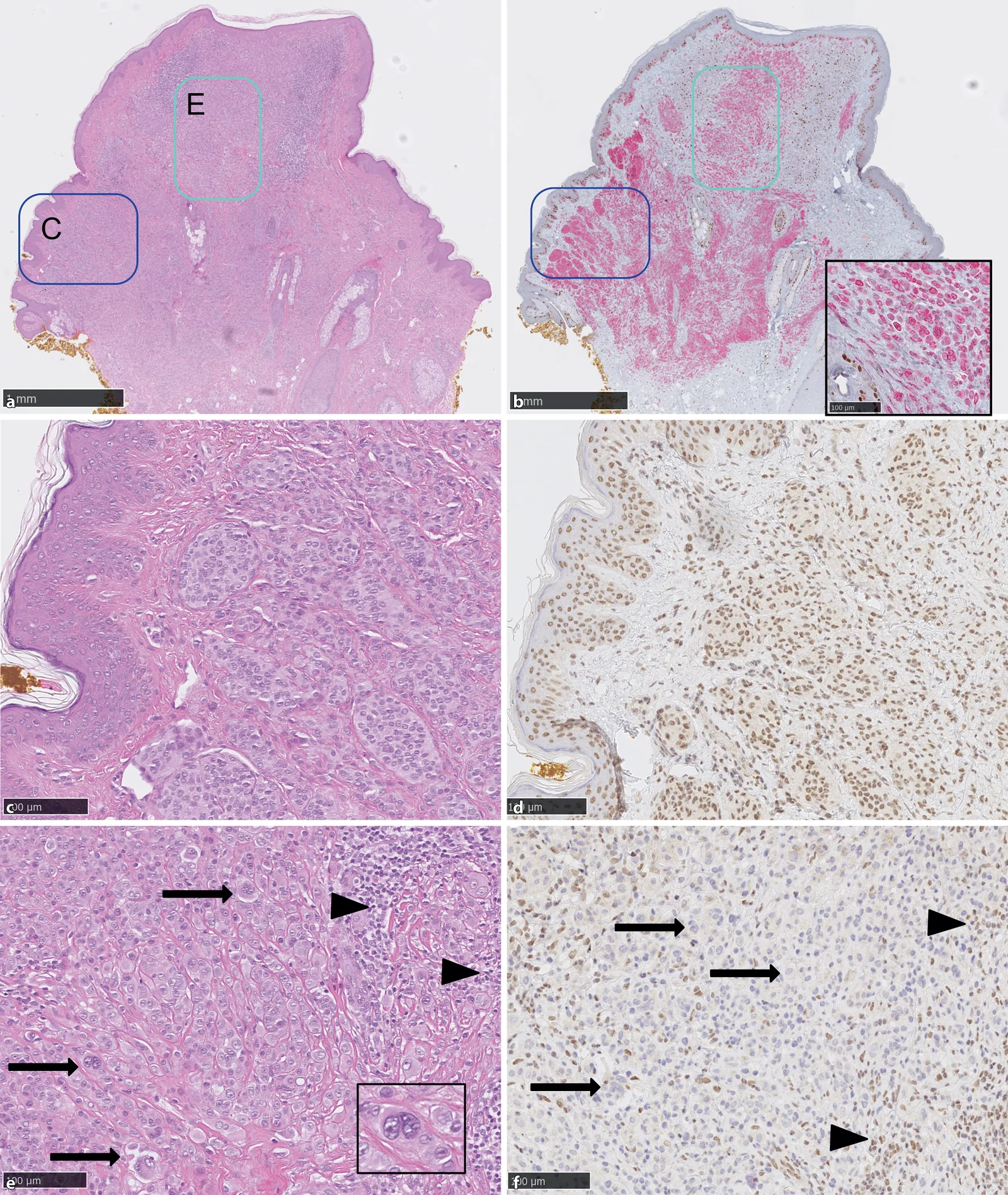
BAP1-inaktiviertes Melanozytom. **a** Übersicht HE: Dermale melanozytäre Läsion mit 2 verschiedenen Zellpopulationen (*blaue* und *türkise* Markierung). Epidermis vorgewölbt nicht involviert (Vergr. 25:1). **b** Immunhistochemische Doppelfärbung Melan-A(*rot*)/Ki67(*braun*): Darstellung der biphasischen Morphologie und Proliferation < 1 % in beiden Tumoranteilen (*Inset*: atypische Zellen ohne Proliferation). **c** Vergrößerung der *blauen* Markierung in **a**: Nävuszellen mit blander Zytologie (Vergr. 300:1). **d** Nukleäre BAP1-Positivität in Nävuszellen (Vergr. 300:1).** e** Vergrößerung der Türkismarkierung in **a**: Große, deutlich atypische, epitheloide Zellen mit viel eosinophilem Zytoplasma und scharfen Zellgrenzen. Kerne vesikulär mit prominenten Nukleolen, teils mehrkernig (*Pfeile* und *Inset*). Lymphozytäres Entzündungsinfiltrat (*Pfeilspitzen*; Vergr. 300:1).** f** BAP1-Immunhistochemie von **e**: Negativität in den atypischen Zellen (*Pfeil*) bei Positivität in Lymphozyten (*Pfeilspitzen*; Vergr. 300:1)

- Histologisch zeigt sich eine dermale Proliferation mit polypoider Silhouette oder Vorwölbung der Epidermis.
- Die Tumorzellen sind groß, epitheloid und besitzen scharf begrenzte Zellmembranen. Das Zytoplasma ist reichlich und eosinophil. Die Zellkerne groß, atypisch, vesikulär und enthalten prominente Nukleolen. Häufig Nachweis einer vorbestehenden Nävuskomponente und eines lymphozytären Infiltrats (Abb. 4).
- Diese Morphologie weist eine deutliche Ähnlichkeit zu Spitz-Läsionen auf und wird in der Literatur auch als BIM mit spitzoider Morphologie beschrieben.
- Zunehmend werden auch seltenere morphologische Varianten beschrieben: kleinzellig-epitheloid, hellzellig, rhabdoid, plasmazystoid und Varianten mit Reed-Sternberg- bzw. Hodgkin-artigen Zellen. Die mitotische Aktivität ist in der Regel gering oder fehlt vollständig [12].

### Immunhistochemie

Diagnostisch richtungsweisend ist der immunhistochemische Nachweis einer *BRAF-*V600E*-Mutation* sowie der Verlust der BAP1-Expression in den großen epitheloiden Zellen bei erhaltener interner Kontrolle in Lymphozyten (Abb. 4f). Selten (5–10 % der Fälle), bei bestimmten Missense-Mutationen, insbesondere außerhalb funktionell relevanter Domänen, kann die Proteinexpression erhalten bleiben. Bei entsprechendem morphologischem Verdacht sollte daher eine molekulargenetische Analyse erfolgen.

### Prognose

BIM zeigen trotz ausgeprägter zytologischer Atypien überwiegend eine günstige Prognose mit indolentem biologischem Verhalten [12, 13].

Obwohl selten, ist eine maligne Transformation möglich. Besonders bemerkenswert sind 2 kürzlich publizierte Fälle mit Lebermetastasen nach vollständiger Exzision. Die Histologie zeigte eine klonale Expansion mit p16-Verlust [28].

BIM werden in der melanozytären Tumorklassifikation der Gruppe 2 zugeordnet und sollten im Gesunden exzidiert werden. Bei Borderlineläsionen, die sich histologisch nicht eindeutig vom Melanom abgrenzen lassen, kann der Sicherheitsabstand bis zu 1 cm betragen [11].

### Differenzialdiagnosen

- Die wichtigste Differenzialdiagnose ist das Melanom. Parameter, welche für eine Melanomdiagnose sprechen oder hinweisend sind: erhöhte Mitoserate ≥ 3/mm^2^, immunhistochemischer Verlust von p16 und Prame-Positivität. Etwa 19 % der BIM sind jedoch ebenfalls Prame-positiv.

Genomische Alterationen mit ≥ 6 Kopienzahlveränderungen, eine komplette 3p-Deletion, Gain von 6p25 (*RREB1*), Verlust von 9p, homozygote *CDKN2A*-Deletion und *TERT*-Promotor-Mutationen sind hinweisend für ein Melanom. Demgegenüber weisen BIM in der Regel einfachere genomische Profile auf [3, 13].

Die Beurteilung eines Borderline-BIM erfordert daher stets eine integrative Beurteilung klinischer, histologischer, immunhistochemischer und molekularer Befunde, da kein einzelnes Kriterium eine absolute diagnostische Sicherheit bietet.

b) Eine weitere wichtige Differentialdiagnose ist der Spitz-Nävus. Im Gegensatz zum BIM weisen Spitz-Tumoren keine *BRAF*-Mutation auf, sondern haben Genfusionen oder eine *HRAS*-Mutationen.

## Schlussbemerkung

Der Vollständigkeit halber sei hier der MITF-Signalweg-aktivierte melanozytäre Tumor erwähnt, da dieser ebenfalls unter der Kategorie MZ gelistet ist. Da die Neoplasie durch eine MITF-Aktivierung getrieben ist, erstaunt es nicht, dass der Tumor immunhistochemisch stark positiv für MITF und melanozytäre Marker wie HMB45 und Melan‑A ist. Ob diese Entität den melanozytären Entitäten zugeordnet werden soll, ist sehr fraglich. Auch PECome, das TTFE3-rearrangierte Nierenzellkarzinom oder das hellzellige Sarkom, bedingt durch Aktivierung des mTor-Signalwegs oder durch Translokation, exprimieren melanozytäre Marker. Dennoch würden wir diese nie den melanozytären Neoplasien zuordnen. Hinsichtlich des MITF-Signalweg-aktivierten melanozytären Tumors verweisen wir deshalb auf aktuelle Literatur [9].

Der Begriff „Melanozytom“ ist kritisch zu betrachten, da dieser in der okulären Pathologie für eine benigne okuläre Läsion verwendet wird. Histopathologisch ist das okuläre Melanozytom mit den stark pigmentierten epitheloiden Zellen nicht von einem PEM zu unterscheiden [23]. Okuläre Melanozytome mit *GNAQ-* oder selten *GNA11-*Mutation sind einem blauen Nävus ähnlich. Diese haben keine „second hits“ und das Risikopotential wird nicht intermediär eingestuft, wie dies beim kutanen MZ der Fall ist.

Die Abgrenzung der verschiedenen kutanen MZ zum Melanom ist manchmal außerordentlich schwierig und macht auch Fachexperten*innen große Schwierigkeiten. Studien zeigen, dass molekulare Zusatzuntersuchungen (NGS) sehr hilfreich sein können für eine bessere Interobserverkongruenz dieser schwierigen Läsionen [3, 17].

## Fazit für die Praxis

- Die 5. Auflage der WHO-Klassifikation der Hauttumoren führt den Begriff der Melanozytome ein.
- Es handelt sich um distinkte Entitäten mit charakteristischer Histopathologie und molekularem Profil.
- Sie zeigen ein intermediäres biologisches Verhalten zwischen Nävus und Melanom.
- Sie besitzen ein meist geringes Progressionsrisiko. Die Exzision sollte jedoch zumindest im Gesunden erfolgen. Je nach Risikoeinschätzung kann der Sicherheitsabstand bis 1 cm betragen.
- Die Abgrenzung zum Melanom ist auch unter Fachexperten*innen manchmal schwierig.
- Molekulare Zusatzuntersuchungen (Next-Generation Sequencing) können bei der Differentialdiagnose Melanom hilfreich sein.

## Data Availability

Alle dieser Arbeit zugrunde liegenden Daten können auf begründete Anfrage beim Korrespondenzautor angefordert werden.

## Abkürzungen

BIM: BAP1-inaktivierte Melanozytome
DPN: WNT-aktivierte, tief penetrierende/plexiforme Melanozytome
MZ: Melanozytome
NGS: Next-Generation Sequencing
PEM: Pigmentierte epitheloide Melanozytome
